# STAT image reporting in a large-scale cohort: types, frequency, and insights from the Tohoku Medical Megabank Brain MRI Study

**DOI:** 10.1007/s11604-025-01800-x

**Published:** 2025-05-15

**Authors:** Naoko Mori, Shunji Mugikura, Yuto Abe, Atsushi Hozawa, Nobuo Fuse, Masayuki Yamamoto

**Affiliations:** 1 Tohoku Medical Megabank Organization, Tohoku University, Sendai, Miyagi Japan; 2 Graduate School of Medicine, Tohoku University, Sendai, Miyagi Japan; 3 Advanced Research Center for Innovations in Next-Generation Medicine, Tohoku University, Sendai, Miyagi Japan; 4 Akita University Graduate School of Medicine, Akita, Akita Japan; 5 Division of Image Statistics, Tohoku Medical Megabank Organization, Tohoku University, 2-1 Seiryo-machi, Aoba-ku, Sendai, Japan; 6 Department of Preventive Medicine and Epidemiology, Tohoku Medical Megabank Organization, Tohoku University, 2-1 Seiryo-machi, Aoba-ku, Sendai, 980-8573 Japan; 7 Department of Integrative Genomics, Tohoku Medical Megabank Organization, Tohoku University, 2-1 Seiryo-machi, Aoba-ku, Sendai, 980-8573 Japan; 8 Department Chair of Education and Training Biochemistry and Molecular Biology, Genetic Epidemiology Research Support, Child Development, Tohoku Medical Megabank Organization, Tohoku University, 2-1 Seiryo-machi, Aoba-ku, Sendai, 980-8573 Japan

**Keywords:** STAT image reporting, Large-scale cohort study, Brain MRI

STAT comes from the Latin word “Statim,” meaning “immediately”. In medical settings, STAT image reporting suggests a potentially life-threatening emergency, requiring prompt reporting of results to the attending physician. The goal is to facilitate rapid diagnosis and treatment initiation, preventing the deterioration of severe conditions [1]. The person performing the STAT image reporting can be the radiological technologist who performed the scanning and the physicians and radiologists who were attending the scanning. In particular, it would be desirable for the radiological technologist who performs the scanning to notice the findings. The importance of STAT image reporting has increased for detecting time-sensitive conditions such as acute cerebral stroke and aortic dissection. In Japan, the use of STAT image reporting is being promoted in order to improve the work style of physicians and radiologists. Recently, the Japan Radiological Society (JRS) and the Japan Association of Radiological Technologists (JART) have established guidelines for STAT image reporting (https://www.jart.jp/news/info/20240305_1117.html). According to these guidelines, typical STAT-reportable findings in brain MRI include hyperintense lesions on diffusion-weighted imaging, which may indicate acute cerebral infarction, encephalitis, encephalopathy, or demyelinating disease. Extra-axial abnormal signal intensities, such as those suggestive of subarachnoid hemorrhage, subdural hematoma, or epidural hematoma, are also considered important findings that warrant immediate attention.

The Tohoku Medical Megabank (TMM) was established to reconstruct the 2011 Great East Japan Earthquake and tsunami. TMM focuses on investigating post-disaster health conditions and aims to contribute to future personalized medicine by establishing a prospective genome cohort and a biobank. Alongside clinical data such as blood pressure and clinical test results from blood and urine, neuropsychological tests and brain MRI are longitudinally collected to investigate the mental health and cognitive function of the large-scale cohort [2]. The interpretation of incidental findings (IFs) of brain MRI was returned to the participants using a results notification system, including urgent notification, with the aim of improving health.

**Fig. 1:**
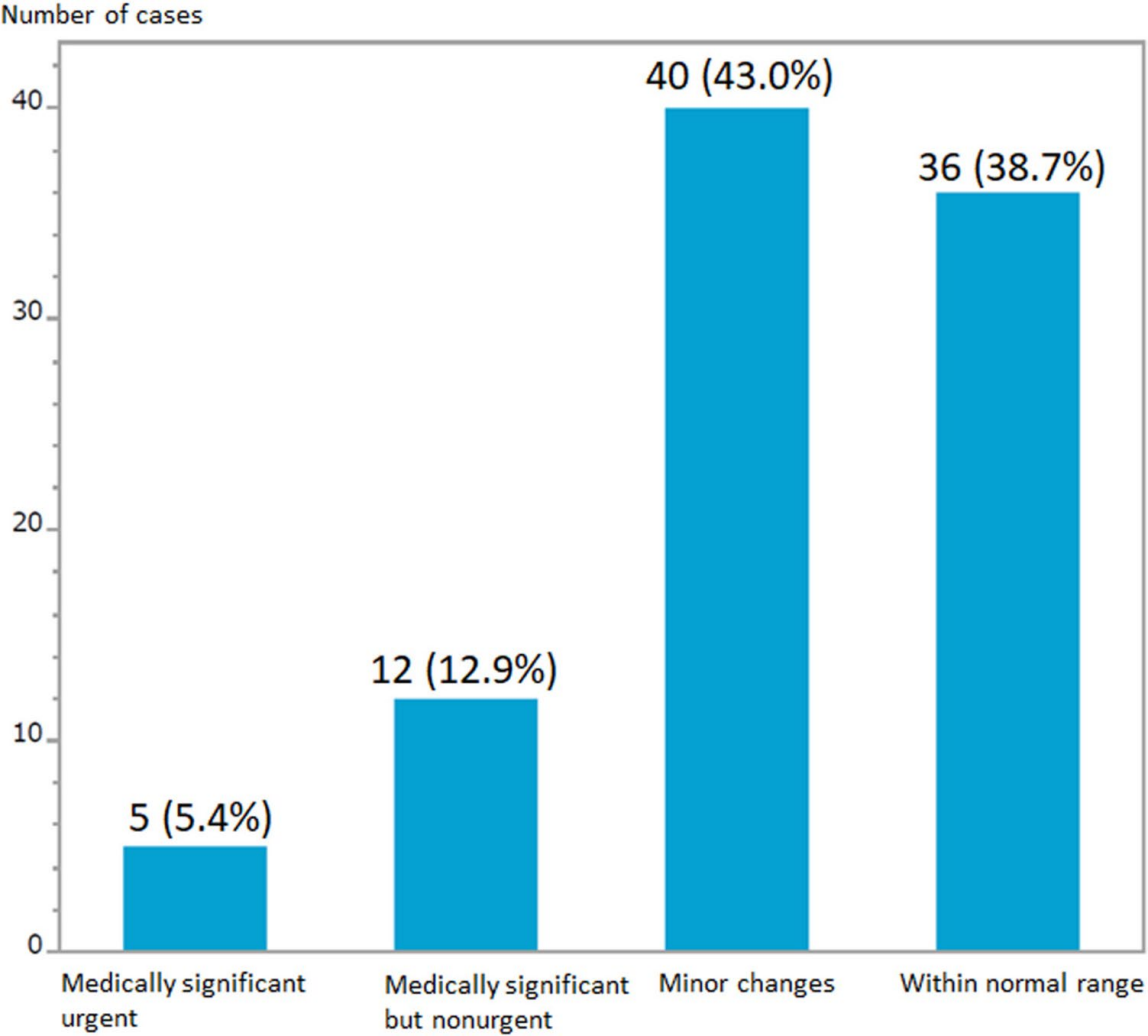
STAT image reporting in Tohoku Medical Megabank brain magnetic resonance imaging (MRI) studies. The findings of brain MRI were classified into four grades, including medically significant urgent, medically significant but non-urgent, minor changes, and within the normal range to notice the examination results to the participants. The radiological technologists reported STAT findings immediately after scanning for 93 cases out of 7403 cases (1.3%), and 5 cases (5.4%) of those were notified to participants as medically significant urgent by radiologists

In the second brain MRI study of TMM conducted from October 2019 to March 2024, 3D-T1 weighted imaging, T2 weighted imaging, susceptibility-weighted imaging, MR angiography, and diffusion tensor imaging were performed on 7,403 participants. Among these, radiological technologists reported STAT image reporting findings to radiologists immediately after the scanning for 93 cases (1.3%). Out of 93 cases, the radiologists promptly reviewed these imaging findings, with 5 cases (5.4%) being notified to participants as medically significant urgent and 12 cases (12.9%) as medically significant but non-urgent. The remaining 76 cases (81.7%) were notified as minor changes or within the normal range after the radiologist compared the present images of the second survey with the previous first brain MR image (Fig. 1). The most common finding reported by radiological technologists was intracranial aneurysms (n = 32, Table 1). Other reported findings included cerebral artery stenosis or occlusion, vascular malformation, acute cerebral infarction, etc. The participants notified as medically significant urgent were intracranial aneurysms (n = 2), cerebral artery stenosis or occlusion (n = 1), and subdural hematomas (n = 2) (Table 1). Meanwhile, other participants with findings such as intracranial aneurysms, cerebral artery stenosis or occlusion, vascular malformations, and acute cerebral infarction reported through STAT image reporting were determined to be non-urgent.

**Table 1:** The types and frequency of STAT image reporting findings and the notification results to participants in Tohoku Medical Megabank brain magnetic resonance imaging study

All (n = 93) (n (%))	Medically significant urgent (n = 5)	Medically significant but nonurgent (n = 12)	Minor changes (n = 40)	Within normal range (n = 36)
Intracranial aneurysm (n = 32)	2 (6.2)	7 (21.9)	14 (43.8)	9 (28.1)
Cerebral artery stenosis/occlusion (n = 15)	1 (6.7)	2 (13.3)	8 (53.3)	4 (26.7)
Vascular malformation (n = 13)	0 (0)	0 (0)	5 (38.5)	8 (61.5)
Acute cerebral infarction (n = 9)	0 (0)	1 (11.1)	5 (55.6)	3 (33.3)
Multiple microbleeds (n = 8)	0 (0)	1 (12.5)	4 (50)	3 (37.5)
Suspicion of meningioma (n = 4)	0	0	2 (50)	2 (50)
Subdural hematoma (n = 2)	2 (100)	0	0	0
Mastoiditis (n = 2)	0	0	0	2 (100)
Suspicion of Moyamoya disease (n = 1)	0	1 (100)	0	0
Others (n = 7)	0	0	2 (28.6)	5 (71.4)

Our results revealed the types and frequency of STAT image reporting by radiological technologists in a large-scale cohort study of TMM brain MRI studies. The hospital brain STAT image reporting includes acute subdural hematoma, intracerebral hemorrhage, epidural hematoma, cerebral infarction, subarachnoid hemorrhage, brain tumor, encephalitis, and encephalopathy. The STAT image reporting in the TMM brain MRI study, a large-scale cohort study, included different types of findings from hospital findings. Still, it included important findings that should be notified to participants who underwent brain MRI. Gibson et al. reported in a systematic review that 2.5% of apparently asymptomatic adults may have serious incidental findings such as malignancy, intracranial aneurysms, or vascular malformations on brain magnetic resonance imaging [3]. The lower frequency of STAT image reporting in our study compared to the systematic review by Gibson et al. may be due to methodological differences. Our analysis focused solely on findings identified and reported by radiological technologists immediately after image acquisition. The overall frequency of potentially significant findings based on full radiological interpretation of all MRI scans is the subject of future investigation. A comprehensive analysis of all imaging data by radiologists would allow for a more accurate comparison with previous studies and a better understanding of the true prevalence of IFs in this cohort. The classification of findings into 'medically significant urgent' and 'medically significant but non-urgent' was determined by radiologists based on their clinical judgment. For instance, intracranial aneurysms with features indicating a high risk of ruptures, such as larger size or irregular shape, were classified as 'urgent'. Currently, there is no standardized definition of 'urgent' findings in the cohort studies like TMM. Therefore, clear criteria and guidelines for this classification will be needed in the future. Further, in this study, participants underwent two brain MRI examinations approximately four years apart, and radiologists interpreted the images from the second examination with reference to the images and reports from the first examination. Based on this comparative evaluation, intracranial aneurysms that were small and unchanged from the previous MRI were categorized as "minor changes." In cases where a lesion suspected of being an aneurysm in the second scan could not be clearly confirmed, it was classified as "within normal range." This approach enabled radiologists to consider both the presence and temporal progression of findings, thereby facilitating classification based on clinical significance.

According to the guidelines of JRS and JART, the development of a STAT image reporting system and ensuring consistency between the findings of radiological technologists and radiologists is essential. Additionally, it is recommended to establish learning systems for radiological technologists focusing on imaging findings that require immediate reporting, along with regular conferences and feedback environments. These efforts aim to improve diagnostic performance and strengthen collaboration between technologists and radiologists. In fact, the STAT Image Reporting Committee has launched the "STAT Image Reporting Learning System" under the supervision of the JRS and the Japanese College of Radiology, in accordance with the “Guidelines for Reporting Imaging Findings (STAT Images) of Life-threatening and Urgent Conditions.” The learning system, hosted by JART, provides structured educational resources for technologists to improve their ability to identify urgent findings (https://www.jart.jp/news/info/20241027_1252.html). Clarifying the decision-making criteria for STAT image reporting is also important for ensuring consistent and reliable identification of urgent findings by radiological technologists. While current judgments rely largely on individual experience, the introduction of standardized assessment protocols and case-based feedback could further improve reporting accuracy and reproducibility of STAT image reporting.

The findings from this large-scale cohort study may help inform the development of future guidelines for STAT image reporting in population-based health screening programs. By identifying the types and frequencies of STAT image reporting findings, cohort studies can provide an evidence-based foundation for determining which imaging abnormalities require prompt medical assessment.
